# Anesthesia for Minimally Invasive Coronary Artery Bypass Surgery

**DOI:** 10.3390/jcdd12060232

**Published:** 2025-06-18

**Authors:** Miranda Holmes, Alexander N. J. White, Luke J. Rogers, Piroze M. Davierwala

**Affiliations:** 1 Department of Anesthesia and Pain Management, Toronto General Hospital, University Health Network, Toronto, ON M5G 2C4, Canada; alex.white@uhn.ca; 2 Department of Anesthesiology and Pain Medicine, University of Toronto, Toronto, ON M5S 1A1, Canada; 3 Division of Cardiovascular Surgery, Peter Munk Cardiac Centre, Toronto General Hospital, University Health Network, Toronto, ON M5G 2C4, Canada; luke.rogers@uhn.ca (L.J.R.); piroze.davierwala@unh.ca (P.M.D.); 4 Department of Surgery, University of Toronto, Toronto, ON M5S 1A1, Canada

**Keywords:** minimally invasive, cardiac surgery, anesthesiology, coronary artery bypass grafting

## Abstract

Minimally invasive coronary artery bypass grafting (MI-CABG) has emerged as a transformative approach to coronary revascularization, offering reduced morbidity, faster recovery and improved cosmesis compared to conventional coronary artery bypass grafting (CABG). Performed without full sternotomy and commonly without cardiopulmonary bypass (CPB), MI-CABG encompasses a variety of techniques. These procedures present unique challenges for the anesthesiologist, necessitating a tailored perioperative strategy. This review explores the anesthetic management of MI-CABG, focusing on preoperative assessment, intraoperative techniques, and postoperative care. Preoperative evaluation emphasizes cardiac, respiratory, and vascular considerations, including suitability for one-lung ventilation (OLV) and the impact of comorbidities. Intraoperatively, anesthesiologists must manage hemodynamic instability, ensure effective OLV, and maintain normothermia. Postoperative strategies prioritize multimodal analgesia, early extubation, and rapid mobilization to leverage the benefits of a minimally invasive approach. By integrating surgical and anesthetic perspectives, this review underscores the anesthesiologist’s pivotal role in navigating the physiological demands of MI-CABG. As techniques evolve and experience grows, a comprehensive understanding of these principles will enhance the safety and efficacy of MI-CABG, making it a viable option for an expanding patient population.

## 1. Introduction

Coronary artery bypass grafting (CABG) has been a fundamental part of coronary artery disease management since the introduction of cardiopulmonary bypass (CPB) in the 1950s and it remains the gold standard for many patients [1]. The vast majority of CABG procedures are performed via a median sternotomy on cardiopulmonary bypass [2]. Over time however, technological advancements in consort with the evolution of surgical and anesthetic technique have allowed surgical revascularization to be performed in a “minimally invasive” fashion.

Although the exact definition of minimally invasive cardiac surgery remains somewhat nebulous, the Society of Thoracic Surgeons [3] defines it as “any procedure not performed with a full sternotomy and CPB support”, while the American Heart Association defines it as “cardiac surgery performed through a small chest wall incision that does not include a full sternotomy” [4].

Minimally invasive coronary artery bypass grafting (MI-CABG) has grown in popularity as it can offer many advantages compared to a conventional CABG procedure. When performed without cardiopulmonary bypass and aortic cross-clamping, it may be associated with a reduced risk of stroke and other neurocognitive complications, kidney injury, blood transfusion, and atrial fibrillation [5,6]. Furthermore, a minimally invasive approach offers better wound healing, a faster recovery and improved cosmesis compared to conventional sternotomy [7].

The success of MI-CABG relies not only on precise surgical techniques but also on the anesthesiologist’s in-depth understanding of the procedure’s critical steps. This article describes the common techniques used in MI-CABG surgery as they are relevant to the anesthesiologist and explores the preoperative, intraoperative, and postoperative anesthetic management including challenges specific to these procedures. By understanding the intricacies of anesthesia for MI-CABG, anesthesiologists play a pivotal role in enhancing patient outcomes and supporting the overall success of the surgery.

## 2. Principles of Minimally Invasive Coronary Bypass Surgery

There is no standardized nomenclature for the different MI-CABG techniques, and considerable institutional variation may exist. We have categorized the different general approaches to MI-CABG with a focus upon aspects that are particularly relevant to the anesthesiologist. Table 1 summarizes these approaches.

### 2.1. Minimally Invasive Direct Coronary Artery Bypass Grafting (MIDCAB)

The MIDCAB technique aims to achieve revascularization of the anterior wall of the left ventricle (LV) by grafting the left internal mammary artery (LIMA) to the left anterior descending artery (LAD) with or without simultaneous grafting of a diagonal artery. It is typically indicated for isolated proximal LAD disease [8]. However, it may also be part of a hybrid approach to multivessel coronary disease, where the LIMA-LAD grafting is combined with percutaneous coronary intervention (PCI) to non-LAD territories [7].

**Figure 1:**
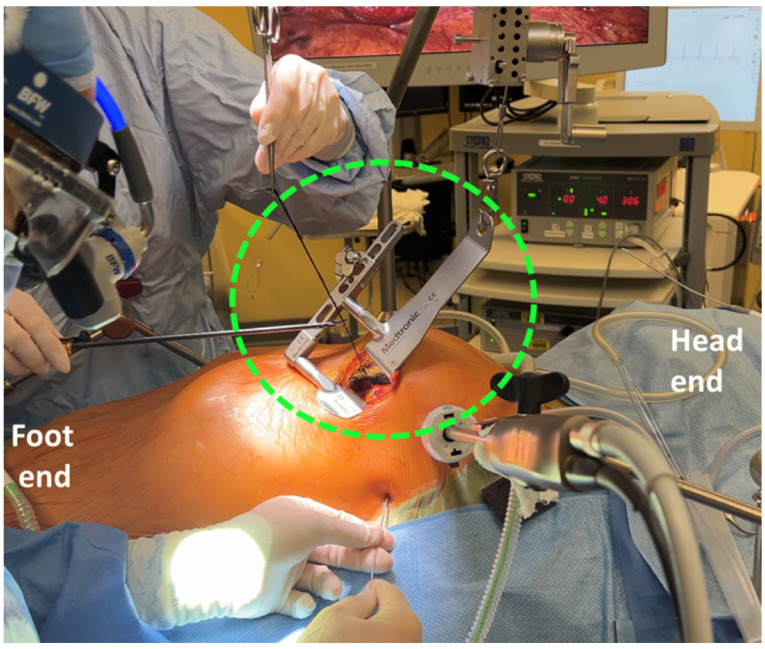
Thoratrak retractor (green hatched circle) elevating the left upper hemi-thorax for exposure and harvest of the left internal mammary artery (LIMA).

A MIDCAB is performed through a left anterior small thoracotomy (5–8 cm), typically in the fourth or fifth intercostal space [9,10]. The LIMA is harvested with the assistance of a rib retractor, such as the Thoratrak (Medtronic Inc., Minneapolis, USA) as shown in Figure 1. Following this, the pericardium is opened, the LAD is identified, and an epicardial mechanical pressure or suction stabilizer is used to stabilize the area at the anastomotic site. The coronary arteriotomy is then performed and the anastomosis is completed, preferably with the use of an intraluminal shunt to maintain coronary perfusion [11]. The procedure is typically performed under direct vision; however, it can be modified with the addition of an endoscopic camera via an additional port site in the left chest to facilitate visualization [11].

Robot-assisted MIDCAB procedures are performed in only a handful of centers worldwide [11]. This technique utilizes three trocars placed longitudinally in the midclavicular line at the second, fourth and sixth intercostal spaces to facilitate harvesting of the LIMA, with the main advantage being avoidance of rib retraction [12]. Conversion to full sternotomy is rare during a MIDCAB, with reported rates of 1–9% [9,13,14].

### 2.2. Multivessel Minimally Invasive Coronary Artery Bypass Grafting (MICS-CABG)

The MICS-CABG is an evolution of the MIDCAB technique that is suitable for patients with multivessel coronary artery disease. The goal is to achieve the same revascularization as a conventional CABG using only a small thoracotomy on a beating heart, and ideally without the need for CPB, although some centers do institute peripheral CPB with or without aortic clamping [15,16].

The left anterior thoracotomy is performed approximately 2–3 cm more laterally than that of a MIDCAB, and exposure is facilitated by the Thoratrak retractor, or a similar device [11,15]. This technique lends itself to an an-aortic (i.e., no aortic manipulation), total arterial approach as additional grafts can most easily be undertaken with an in situ right internal mammary (RIMA) to LAD or left circumflex (LCx) artery through the transverse sinus or as composite grafts undertaken sequentially off the LIMA [16,17]. This can also be undertaken with a radial artery or saphenous vein if desired. This is the preferred technique in our center, and it avoids the requirement for a proximal anastomosis onto the ascending aorta. If the surgical approach involves proximal anastomosis onto the ascending aorta, then a partially occluding side-biting clamp is required.

**Figure 2:**
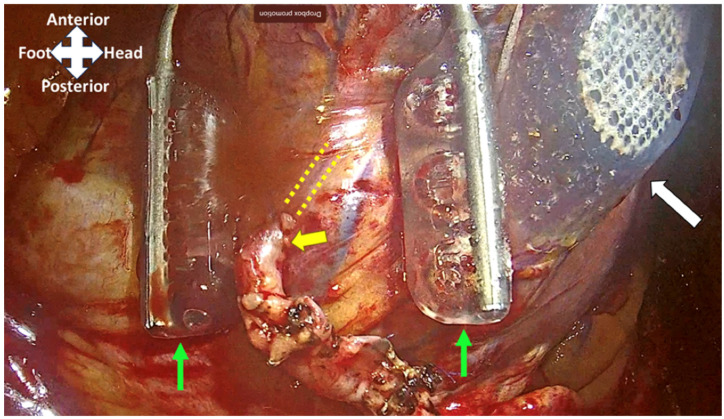
Completed RIMA-OM anastomosis (yellow arrow). Heart positioner (white arrow) and suction stabilizer (two green arrows) exposing and stabilizing the OM (dashed parallel yellow lines) anastomotic site.

**Figure 3:**
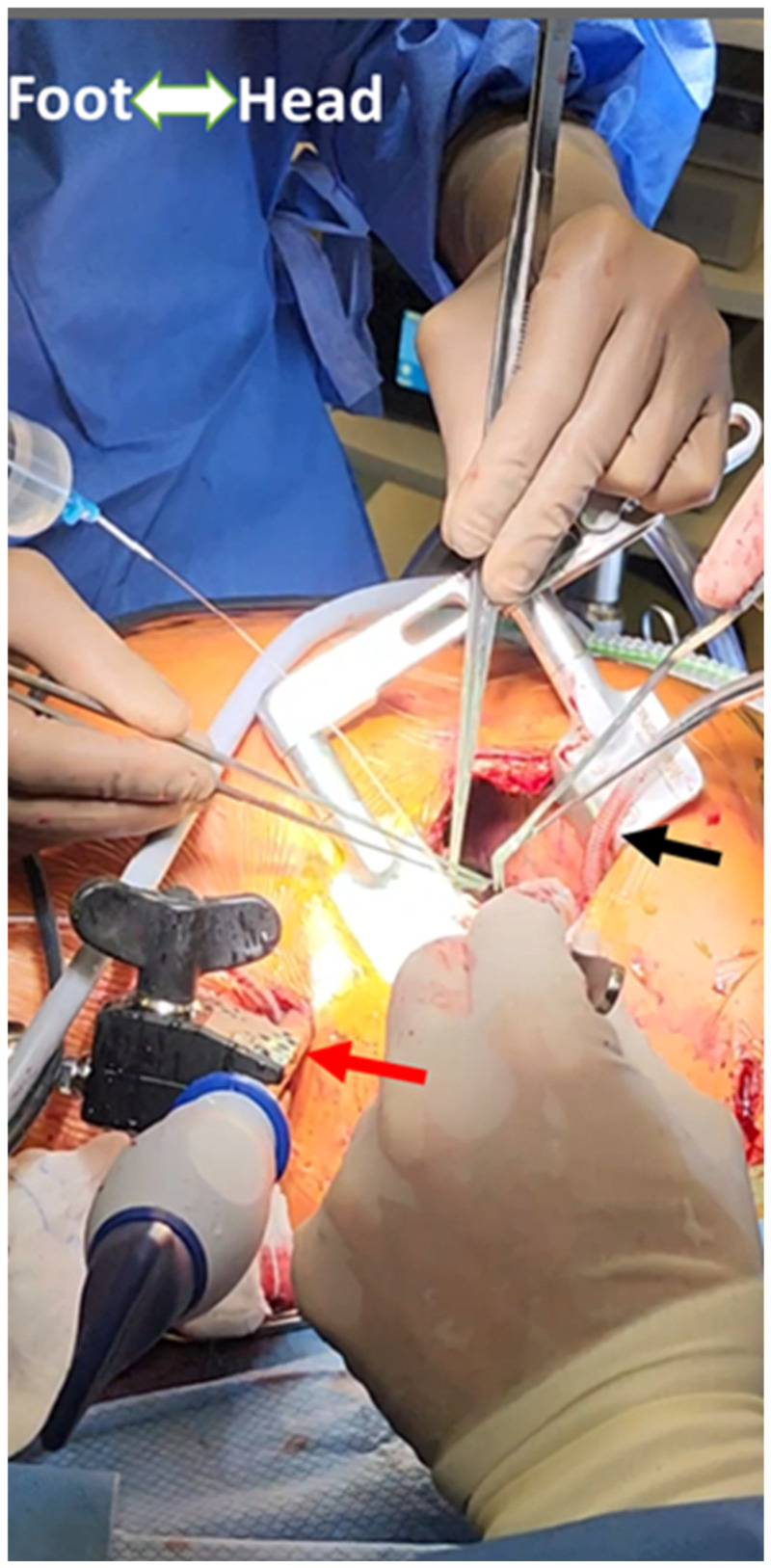
External setup for grafting the lateral/inferior wall vessels in MICS-CABG illustrating the entry sites for the heart positioner (black arrow) and suction stabilizer (red arrow).

To expose the distal anastomosis sites on vessels such as the obtuse marginal or posterior descending artery (PDA) branches of the LV, a specialized heart positioner is applied to the lateral or inferior wall [15]. The suction stabilizer is then used to stabilize the target vessel at the site of the anastomosis, which is performed in either a side-to-side or end-to-side fashion, depending on whether a sequential or single graft is performed. Figure 2 shows the heart positioner and suction stabilizer with a completed anastomosis. Figure 3 shows the external setup for grafting the lateral/inferior wall vessels in MICS-CABG.

### 2.3. Total Endoscopic Coronary Artery Bypass (TECAB)

The TECAB technique aims to perform coronary revascularization through only endoscopic port sites [11], entirely robotically and therefore eliminating the need for even a small thoracotomy [18]. While initially developed for single-vessel LIMA-LAD grafting as described above, it has also been used to facilitate multivessel grafting [7]. The first port into the left hemithorax is made in the same manner as in video-assisted thoracic surgery (VATS) with a Veress needle to insufflate CO_2_ at 8–10 cmH_2_O, and the additional ports are added under direct visualization [12]. The LIMA harvest is undertaken in an identical fashion to a robotic MIDCAB and often also includes robotic harvest of the RIMA. The additional ports are utilized for a robotic epicardial mechanical stabilizer and additional instruments as the coronary targets require [12]. The anastomosis has historically been undertaken by the C-Port Flex A (Aesculap, Tutlingen, Germany) distal anastomotic stapler [19], although a recall issued in 2018 means that all anastomoses must now be sutured primarily with the robot. Although comparable in safety to other coronary bypass grafting methods, the TECAB can have a lengthy procedural time [11].

### 2.4. Cardiopulmonary Bypass-Assisted Minimally Invasive Coronary Artery Bypass Grafting

The MIDCAB, MICS-CABG, and TECAB techniques described above are ideally performed without the need for CPB, adhering to the strictest definition of “minimally invasive” cardiac surgery. There may however be considerable hemodynamic disturbance associated with retraction of the heart causing myocardial ischemia from hypotension, vessel manipulation, and/or hypoxemia from OLV. In high-risk cases, the prophylactic institution of CPB can be used to minimize this physiological derangement. If required, this is performed peripherally through cannulation of the common femoral artery (CFA) and vein (CFV).

Grafting can be performed on a beating heart with CPB providing hemodynamic and respiratory support. In cases when aortic cross-clamping may be necessary, this can be achieved through two additional incisions in the left thorax to insert a Chitwood clamp and cardioplegia delivery system.

Alternatively, the use of an endovascular aortic occlusion balloon (IntraClude, Edwards Inc., Irvine, CA, USA) may be placed either in the contralateral CFA or a sidearm of the aortic cannula. The device is advanced into the ascending aorta under transesophageal echocardiogram (TEE) and hemodynamic guidance from the femoral and right radial arterial lines [20]. The balloon is inflated, occluding the ascending aorta and allowing cardioplegia delivery into the aortic root.

## 3. Preoperative Assessment

As with traditional CABG, the anesthetic evaluation of patients undergoing MI-CABG involves thorough review of the patient’s medical history, a physical exam, laboratory investigations, and imaging. However, there are specific considerations to the minimally invasive approach that are of special importance.

### 3.1. Cardiac

#### 3.1.1. Coronary Arteries

A thorough understanding of the timing and extent of the patient’s coronary artery disease is essential. Patients who have an acute LAD occlusion or iatrogenic LAD dissection are not considered appropriate for MI-CABG [21]. Similarly, patients with acute coronary syndrome and concomitant hemodynamic instability are not considered suitable [21]. However, patients requiring urgent surgery who are hemodynamically stable (e.g., non ST-elevation myocardial infarction, unstable angina) may be candidates for MI-CABG.

A small LIMA (<1.5 mm in diameter) or an intramyocardial LAD were previously considered to be contraindications to MI-CABG [22], but as more experience has been gained in the field, these may no longer be absolute restrictions [7].

Understanding which vessels are being grafted is important to help predict the extent of hemodynamic instability during the procedure. Grafting of the LAD is usually feasible without displacement of the heart, but if the LCx and/or RCA territories are involved, significant subluxation will be required and the likelihood of hemodynamic instability will increase [23]. The need for RCA grafting in the absence of a suitable PDA target, or circumflex territory grafting in the absence of a suitable obtuse marginal target could make MICS-CABG prohibitively challenging [15].

It is also important to understand if the procedure is being performed as a part of a hybrid approach. In cases where PCI is planned after surgery, only partial revascularization will occur during the operation. Postoperative ischemia is therefore more likely and anesthetic and intensive care teams should be judicious in recognizing this early if it occurs.

#### 3.1.2. Left Ventricular Function

Historically, impaired LV function was considered a significant contraindication to MI-CABG procedures. Over time, however, it has been demonstrated that in fact it can be safely and successfully performed in patients with impaired LV function [17,21,24]. Caution should still be taken in patients with a left ventricular ejection fraction (LVEF) < 20%, severe LV dilation (LVEDD > 55 mm), or hemodynamic instability [25,26]. Cardiomegaly can also be identified on chest x-ray, and some centers use a cardiothoracic ratio of >50% as a cutoff for suitability [25].

A thorough echocardiographic evaluation of LV wall motion is important. Knowledge of preexisting regional wall motion abnormalities (RWMAs) is helpful when analyzing the intraoperative echocardiogram to determine whether any new RWMAs arise.

#### 3.1.3. ECG

A preoperative ECG should be analyzed to identify conduction abnormalities and other dysrhythmias. Bradyarrhythmias and conduction blockade can be exacerbated during the conduct of surgery, and emergency pacing can be difficult to establish. Mechanical manipulation of the heart during the procedure can also trigger ventricular tachyarrhythmias, which can be challenging to address intraoperatively. Although conduction abnormalities are not an absolute contraindication, the anesthesiologist should be aware of them and consider approaches to intraoperative pacing.

#### 3.1.4. Valvular Function

While the presence of concomitant valvular disease is not in and of itself a contraindication to MI-CABG, intervention on the valve may be necessary, which likely includes a sternotomy and CPB.

In patients with severe aortic stenosis, a recent case series has demonstrated the feasibility of a staged approach in which patients received their MI-CABG procedure followed shortly thereafter by a transcutaneous aortic valve implantation (TAVI) [27].

The presence and degree of aortic insufficiency should be delineated, as it may preclude the use of CPB-assisted MI-CABG with an arrested heart due to unreliable cardioplegia and LV distension.

#### 3.1.5. Pericardium

The presence of inflammatory pericardial disease or pericardial adhesions from previous surgery or radiation are a relative contraindication [28]. MI-CABG procedures have been safely performed as part of redo coronary artery bypass grafting [29].

### 3.2. Respiratory

#### 3.2.1. Suitability for One-Lung Ventilation

A cornerstone of minimally invasive cardiac bypass grafting is the ability to operate through a small thoracotomy, which relies on one-lung ventilation (OLV) [15]. Patients are positioned supine, with a slight lateral tilt which may increase the risk of desaturation on OLV [30,31]. However, a semi-lateral decubitus position has been shown to be equally protective against desaturation on OLV compared to a lateral decubitus position [32,33]. Periods of reduced cardiac output during retraction and manipulation of the heart also predispose the patient to desaturation [17,34]. In otherwise healthy patients, significant desaturation during MI-CABG surgery on OLV is rare [33]. Preoperative evaluation should therefore identify additional risk factors for hypoxemia on OLV, the presence of which may warrant additional testing.

A history of chronic lung disease places patients at risk of hypoxemia on OLV, and it may be prudent to quantify the degree of disease with pulmonary function testing and arterial blood gas analysis [30]: patients with low PaO_2_ are generally considered less suitable for OLV [31,35]. A retrospective analysis of patients undergoing robotic TECAB demonstrated that patients at higher risk of conversion to sternotomy had significantly lower forced vital capacity (FVC) and forced expiratory volume in 1 s (FEV1); however, reduction in these parameters did not have any significant effect on clinical outcome [20]. The presence of obesity (BMI > 30) and increased age are other risk factors for hypoxemia on OLV [31], which are common in the cardiac surgical patient population. There are no absolute cutoff values that would preclude a patient from having surgery, but the anesthesiologist should take a gestalt approach to determine overall suitability for OLV.

Interestingly, a small retrospective study of patients with chronic obstructive pulmonary disease (COPD) undergoing minimally invasive valve surgery using OLV demonstrated reduced postoperative complications, ICU length of stay, and time to hospital discharge [36]. This suggests that although patients with chronic lung disease may prove to be more challenging to manage in the operating room, provided they can maintain a physiologic saturation during the operation, a minimally invasive approach may be an overall benefit in reducing early postoperative respiratory complications.

A detailed airway examination, chest x-ray, and CT scan interpretation can help determine the ease of DLT placement and may influence the choice of lung separation technique.

#### 3.2.2. Pleural Disease

Pleural adhesions may impede minimally invasive surgical access [37]. Patients with a history of prior thoracic surgery, chest trauma, empyema, tuberculosis, bronchiolitis obliterans, or systemic lupus erythematosus are at risk of having densely adherent pleura. The presence of one or more of these may increase the likelihood of bleeding and conversion to sternotomy.

#### 3.2.3. Pulmonary Hypertension

It is crucial to assess any level of pulmonary hypertension and resulting right ventricular (RV) dysfunction. All patients should undergo a preoperative echocardiogram, which should provide an estimate of the right ventricular systolic pressure (RVSP), as well as information on the size and systolic function of the RV.

While pulmonary hypertension is not an outright contraindication to MI-CABG, OLV can temporarily raise the RV afterload by increasing pulmonary vascular resistance. This is compounded by transient reductions in contractility and increases in left atrial pressure with heart retraction, potentially leading to RV failure and hemodynamic collapse.

### 3.3. Vascular

Given the LIMA-LAD graft is the lynchpin of MIDCAB and many MICS-CABG procedures, the presence of hemodynamically significant left subclavian artery stenosis is a contraindication to MI-CABG, as this can result in subclavian steal syndrome [15,38]. For the same reason, the presence of a left arm arteriovenous (AV) fistula is also a contraindication [15].

An evaluation for the presence of atheromatous disease, aneurysms, dissections, stents or grafts, and femoral artery caliber is essential, especially if peripheral CPB is planned [39,40]. CT angiography can be performed to screen for the presence of prohibitive vascular disease [30,41].

The presence of an inferior vena cava (IVC) filter is traditionally thought to be unsafe to use in conjunction with peripheral venous cannulation for CPB, with theoretical risks of migration, deformation, or fracture of the stent, as well as resistance when advancing the cannula [42]. However, there are case reports of successful femoral venous CPB in the presence of IVC filters without complication [42,43].

### 3.4. Gastrointestinal

Intraoperative TEE is used in many patients having MI-CABG. Conditions such as esophageal webs or strictures, tumors, lacerations, diverticula, or active upper gastrointestinal bleeding are absolute contraindications to TEE [44]. Relative contraindications include a history of radiation to the head and neck or mediastinum, previous surgery of the upper gastrointestinal tract, esophageal varices, active peptic ulcer disease, and hiatal hernia [44]. In the presence of any of these risk factors, a discussion with the surgical team is warranted about whether or not the operation can be performed safely without TEE. In our institution, the use of TEE in patients with normal biventricular function undergoing a single-vessel MIDCAB is not routine, but it is employed in all multivessel MICS-CABG procedures.

### 3.5. Body Habitus

#### 3.5.1. Musculoskeletal

Chest wall deformities can make minimally invasive surgical access difficult, particularly if the thoracic cavity is small [30]. Pectus excavatum is considered a relative contraindication, but MIDCAB procedures have been performed in these patients safely [45]. Kyphoscoliosis can limit the ability to position the patient appropriately to gain sufficient surgical access [46].

#### 3.5.2. Obesity

The presence of obesity can make the conduct of direct vision MI-CABG a challenge for several reasons. Surgical access to the chest may be difficult, particularly in patients with large breasts [47]. Most often, the surgeon has access to the heart and the LIMA through a long tunnel, which makes exposure and visualization suboptimal. Additionally, visualization of the LIMA and coronary artery targets can be challenging in the presence of excessive fatty tissue within the chest and can lengthen the duration of surgery [48]. Obesity can also hinder the ability to properly position patients, and they may be less likely to tolerate OLV.

**Figure 4:**
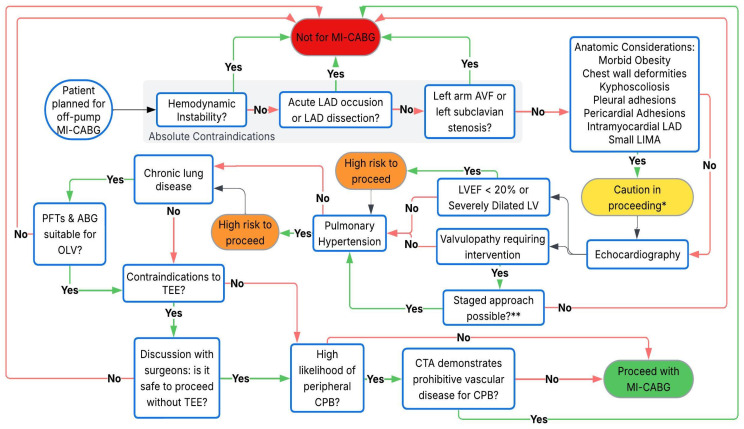
An algorithm to help guide preoperative decision-making in patients considered for off-pump MI-CABG. This is intended as a framework; actual clinical decisions may vary based on individual surgeon preference, type of procedure, and institutional protocols. * May pose technical challenges depending upon surgeon experience and type of procedure (direct vision vs. endoscopic). ** A staged TAVI approach may be feasible in patients with concomitant aortic stenosis. MI-CABG = minimally invasive coronary artery bypass grafting, LAD = left anterior descending coronary artery, AVF = arteriovenous fistula, LIMA = left internal mammary artery, LV = left ventricle, LVEF = left ventricular ejection fraction, PFT = pulmonary function test, ABG = arterial blood gas, OLV = one-lung ventilation, TEE = transesophageal echocardiography, CPB = cardiopulmonary bypass, CTA = computed tomography angiogram.

Despite these intraoperative challenges, obese patients may benefit the most from a minimally invasive approach in the long term because they are at risk of delayed wound healing and are more prone to wound infections with a sternotomy [48]. Table 2 summarizes the indications and contraindications to MI-CABG procedures. A guide to perioperative decision making is shown in Figure 4.

## 4. Intraoperative Management

### 4.1. Setup

#### 4.1.1. Operating Room Equipment

The operating room is set up in a similar fashion to that used in conventional cardiac surgery. A heart–lung machine must be present and primed with a perfusionist present throughout the surgery in the event that the need for emergency conversion to CPB arises. Additional equipment that must be available includes a TEE machine and probe, intraoperative cell salvage, and a sternotomy saw.

#### 4.1.2. Airway Equipment

In order to facilitate surgery, OLV is almost always required in order to keep the left lung out of the surgical field. This is most often carried out using a left-sided double-lumen endobronchial tube (DLT) under bronchoscopic guidance with a pediatric bronchoscope. Alternatively a single lumen endotracheal tube may be used with a bronchial blocker.

The advantages of a DLT over a bronchial blocker include relative ease of placement, the ability to easily suction and perform bronchoscopy in each lung field, and security of placement with a lower likelihood of intraoperative displacement [49]. However, the use of DLTs can increase the likelihood of tracheal and bronchial injury, as well as throat discomfort [49,50].

A bronchial blocker may be the method of choice in the case of a difficult airway. It may also be advantageous if the patient is to remain intubated at the end of the procedure, because no tube exchange is necessary before ICU transfer.

#### 4.1.3. Monitoring

In conventional cardiac surgery, the anesthesiologist’s direct visualization of the surgical field is one of the most important monitors. In MI-CABG, however, the limited visibility due to smaller incisions and specialized instruments reduces this ability. Consequently, extra vigilance is required, and anesthesiologists must maintain continuous communication with the surgical team, particularly during periods of physiological disturbance.

Accurate ECG monitoring is essential. The use of a left thoracotomy may prevent the precordial (V) lead from occupying its standard position, and it is important to understand what myocardial territory is being displayed. Myocardial ischemia can occur during revascularization and often the first sign is ST segment changes on the ECG, and it is important to both recognize and localize it.

The nature of MI-CABG makes internal defibrillation challenging and often impossible. External defibrillator pads are necessary for all patients. One is placed over the right pectoralis muscle below the nipple, and the other over the left scapula making sure not to obstruct a possible median sternotomy if required emergently. If defibrillation is necessary during OLV, the left lung may need to be temporarily re-expanded to allow for better conductivity between pads.

Invasive arterial blood pressure monitoring is typically carried out through the right radial artery, keeping the left radial available as a surgical conduit. If an endoballoon is used, both a right radial arterial line and either a left radial, brachial, or femoral arterial line are necessary to detect endoballoon migration and occlusion of the brachiocephalic trunk [51]. If the endoballoon migrates distally, then the right radial arterial pressure waveform will be lost on occlusion of the brachiocephalic trunk, thus necessitating the afterload to be increased to force the balloon back proximally.

Central venous pressure monitoring is carried out through a right or left internal jugular central venous catheter. Pulmonary artery pressure monitoring with a Swan–Ganz catheter may be useful in detecting cardiac output changes quickly during surgical manipulation [52] and can be useful in certain high-risk cases. However, many anesthesiologists now opt for alternative methods that provide similar information without the added risks of pulmonary artery catheterization. If intraoperative pacing is likely to be required, a pacing Swan–Ganz catheter can be placed.

Cerebral oximetry is not routinely used in all patients; however, it can be particularly useful in cases with higher risks of cerebral ischemia. Additionally, when beating-heart peripheral CPB is used with insufficient gas exchange in the lungs, there may be differential hypoxia in the upper parts of the body (including the brain and coronary arteries). Cerebral oximetry may help detect early signs of Harlequin syndrome [53].

Depth of anesthesia monitoring and train-of-four monitoring are useful in order to ensure appropriate depth of anesthesia and neuromuscular blockade, as inadvertent movement during the procedure can have deleterious consequences [40,54].

#### 4.1.4. Temperature Management

Temperature management in MI-CABG can be challenging and differs from conventional CABG procedures where CPB is used for temperature regulation. Maintenance of normothermia should begin in the preoperative area with the application of a forced air warmer. Maintaining a relatively warm operating room ambient temperature further helps minimize heat loss. Once the patient is adequately prepped, they should be draped as quickly as possible to minimize the evaporative cooling effect.

Intraoperatively, the surface area of the patient available for forced air warming is limited. Sterile forced air warmers are available and can be placed over the lower body once conduits have been satisfactorily harvested. Intravenous (IV) fluid warmers and underbody warming mats are used intraoperatively.

#### 4.1.5. Medications

Tranexamic acid (TXA) has not been extensively studied in MI-CABG, particularly when compared to its use in conventional on- and off-pump CABG patients. TXA is not routinely administered in MI-CABG cases, unless CPB is being used, where its role in reducing blood loss may be more relevant.

Vasopressor infusions (typically norepinephrine and vasopressin) must be available. Because of the rapid hemodynamic changes that can occur, vasoactive medications must also be available in bolus form. In our institution we typically prepare syringes with diluted concentrations of phenylephrine (40 mcg/mL), norepinephrine (4 mcg/mL), and epinephrine (4 mcg/mL).

The use of an initial load of IV milrinone (25–50 mcg/kg) over 20–30 min can help facilitate distal anastomoses by reducing cardiac size [26], especially in patients with impaired LV function or heart failure with preserved ejection fraction. The use of preoperative levosimendan for 24 h before surgery can be considered in patients with impaired LV function, as it has shown both short- and long-term benefits in a retrospective analysis in a general cardiac surgical population [55]. However, levosimendan is not currently licensed for use in many countries including North America, Australia, and the United Kingdom, and IV milrinone is a suitable alternative.

Postoperative nausea and vomiting prophylaxis can be incorporated as part of the care bundle for patients planned for early extubation, as recommended in a recent systematic review of enhanced recovery after minimally invasive valve surgery [56].

## 5. Induction and Maintenance of Anesthesia

The induction of anesthesia is largely similar to that of conventional cardiac surgery, as the fundamental goals remain consistent and the technique is tailored to the individual patient. Keeping the heart rate relatively slow and avoiding an overly contractile myocardium is helpful for grafting [57]; a short acting narcotic infusion (remifentanil or sufentanil) may be useful to help facilitate these conditions and will have the additional beneficial effect of helping to inhibit respiratory drive in the setting of OLV and often-associated hypercarbia. The patient is intubated with a DLT, or a single lumen tube and bronchial blocker. The DLT position is confirmed bronchoscopically. If a bronchial blocker is being used, it is often placed after the patient is in their final position because of the high likelihood of displacement while the patient is being moved. Lung deflation can be suboptimal using a bronchial blocker. The inner diameter is very narrow, reducing the efficacy of passive deflation as well as suctioning of the lung. It is therefore important to ventilate the patient with 100% oxygen for a short time, and then to turn off the ventilator until the patient is at end expiration before inflating the bronchial blocker in order to more effectively deflate the lung.

### 5.1. Positioning

For the majority of MI-CABG procedures, access via a small left thoracotomy is optimized by having the patient positioned supine with a 30° right lateral tilt. This is usually achieved by inflating a pressure bag under the left scapula, granting greater exposure of the left hemithorax [58]. The left arm is often tucked at the side. In some cases it may improve operating conditions to have it abducted above the head, although there have been case reports of brachial plexus injury in this position [59,60]. If a left radial graft is being taken, then the arm is initially placed on a padded armboard and abducted. When the conduit harvest is complete, the arm is wrapped and placed in its final position. The right arm is also tucked at the side. Care must be taken to ensure the neck is in a neutral position.

If a robot is being used, the anesthesia workstation must be positioned far enough away from the table that it does not interfere with the robot arms after docking [61]. Care must be taken to avoid the robot arms contacting the patient’s head if the surgeons are working near the diaphragm [61]. Placing some padding or turning the patient’s head slightly right can protect against this [61]. Some centers advocate for the right arm to be slightly (<30°) abducted rather than tucked at the side, because the lateral tilt of the patient can cause compression of the right arm by the abdomen [12].

The position of the DLT or bronchial blocker should be rechecked after the patient is properly positioned.

### 5.2. Desaturation

Desaturation during MI-CABG can occur, and initial measures include ensuring proper positioning of the DLT or bronchial blocker, ensuring airway patency, and adjusting the ventilator settings and FiO_2_. While the insufflation of oxygen and the use of CPAP to the left lung during can improve oxygenation, our experience suggests that this approach significantly obstructs the surgical view.

Desaturation often occurs alongside hypotension and low cardiac output. In these cases, addressing the hemodynamic disturbance (e.g., fluid resuscitation, vasopressor and inotropic support) often resolves the desaturation. If these measures fail, two-lung ventilation may be necessary. The surgeon can pack the left lung posteriorly to maintain proper exposure while both lungs are ventilated [15].

### 5.3. Transesophageal Echocardiography

Initial assessment involves a comprehensive examination including evaluation of ventricular function in the context of tolerating positioning for off-pump grafting as described above and the presence of preexisting RWMAs. Valvular function should be assessed and any unexpected findings that may alter the surgical plan, if present, should be identified. Assessment for any intracardiac shunts should be undertaken especially in the context of potential elevated right ventricular and right atrial pressures with OLV and flow reversal of a left–right shunt. TEE can be used to assist with advanced hemodynamic monitoring including assessment of ventricular filling and forward stroke volume using the velocity time integral and area through the left ventricular outflow tract. If the use of CPB is necessary, TEE can guide percutaneous cannula placement via the femoral vessels and assist with the inflation and maintenance of the position of the endoballoon within the ascending aorta [62].

Ventricular function and RWMAs are observed throughout, particularly while distal anastomoses are being performed. RV function and right ventricular systolic pressure (RVSP) can be monitored during periods of OLV given that direct visualization is not possible. The area of the LV where the heart positioner is applied will appear hypo- or akinetic. Nevertheless this should be communicated to the surgeon.

## 6. Conduct of Surgery

### 6.1. Conduit Harvesting

After appropriate positioning and placement of external defibrillator pads, the patient is prepped and draped. The left anterior small thoracotomy is made. Prior to entering the pleura, the left lung is deflated and OLV of the right lung is commenced. The LIMA is harvested following exposure with the retractor. If a radial artery is being used, this is harvested simultaneously. When the distal end of the LIMA is ready to be separated, and the other conduits are ready, IV heparin is administered. The target activated clotting time (ACT) for MI-CABG procedures is variable, although most aim for an ACT > 300 s and many > 350 s, which is typically achieved with 150–250 IU/kg of heparin. If CPB is planned, then the dose of heparin is increased to achieve an ACT > 480 s.

If a robot is being used, a left-sided capnothorax is created to expand the working space. Insufflation pressures between 10–15 mmHg are used. Hypotension during this phase can occur and is typically treated with intravenous fluids. Minute ventilation of the right lung should be increased to combat hypercapnia. If the capnothorax is not tolerated, the surgical team should be alerted. Insufflation pressures can be decreased, and if necessary the chest can desufflated to allow time for fluid resuscitation and vasopressor therapy to be commenced before reinstituting the capnothorax [63].

### 6.2. Grafting

The most hemodynamic instability occurs during coronary artery grafting, when the distal anastomoses on the lateral and inferior LV walls are being performed. Hemodynamic changes during grafting in conventional off-pump CABG (OP-CABG) surgeries have been well-studied, but less so in MI-CABG procedures.

At this time there may be periods of transient reductions in blood flow and it is important to maintain coronary perfusion pressure while avoiding increases in contractility and heart rate. Surgical conditions improve at lower heart rates, therefore preventing tachycardia is essential. Ensuring adequate analgesia with potent short-acting opioids may be useful. Esmolol may also be used but this must be carried out carefully, because lowering cardiac output excessively could compromise tissue perfusion and precipitate pulmonary edema. Norepinephrine and vasopressin are preferable to maintain systemic pressure over agents with more inotropy and chronotropy. IV fluids may be used to increase the stroke volume; however, placing the patient in Trendelenburg position is another method of transiently augmenting filling pressures and stroke volume during periods of instability without contributing to the net fluid balance and tissue edema.

The distal anastomoses of the grafts are performed with the use of a mechanical epicardial stabilizer. In conventional OP-CABG surgery, application of the stabilizer for the LIMA-LAD anastomosis results in a very mild decrease in mean arterial pressure (MAP), accompanied by a modest decrease in cardiac output and increased atrial and ventricular filling pressures [64]. Therefore, the hemodynamic changes during the LIMA-LAD anastomosis are not usually clinically significant, especially in patients with preserved ejection fractions because the heart does not need to be rotated significantly to expose the LAD target [65,66]. Occasionally the stabilizer may compress a small branch vessel, resulting in local ischemia manifested by ST changes, which should be brought to the attention of the surgical team [67]. Grafting the diagonal before revascularizing the LAD may result in sudden hemodynamic instability due to compression of the LAD by the stabilizer, particularly when the site of the anastomosis is adjacent to the LAD.

The use of an intracoronary shunt is common practice to minimize the risk of myocardial ischemia while performing the anastomosis. It is a device temporarily inserted into the coronary artery to maintain distal perfusion while the vessel is being grafted. If ST segment changes or arrhythmias occur during this period, it may be a result of shunt malposition, and the surgeons should be alerted in order to reposition if necessary.

If multivessel grafting is being used, then the heart is retracted within the chest to expose the targets. The addition of 10–12 cmH_2_O of positive end-expiratory pressure (PEEP) to the right lung, along with a 1:1 inspiratory to expiratory ratio, can help assist in bringing the heart slightly closer to the thoracotomy, facilitating coronary anastomosis [15].

For CPB-assisted procedures, the patient is weaned from CPB following the completion of grafting. Reinflating the left lung temporarily before coming off CPB can help optimize RV function [30].

### 6.3. Hemodynamic Management During Grafting

There is much more hemodynamic embarrassment with multivessel grafting compared with a single LIMA-LAD anastomosis. Ensuring adequate intravascular volume status and administering vasopressors to target a slightly elevated MAP during heart positioning can mitigate profound hypotension and reduced stroke volume. These strategies can be more effective if used proactively rather than reactively. In our center we use a combination of Trendelenburg positioning and intravenous crystalloid limiting fluid administration to 2–3 L to allow adequate preload while minimizing pulmonary congestion. It can be helpful to start or increase the rate of a vasopressor infusion, but we find that the rapidity and duration of hemodynamic disturbances usually require bolus doses to be adequately addressed. We start with intermittent doses of 80–200 mcg of phenylephrine, and if these are ineffective we will use boluses of 4–16 mcg of norepinephrine. Dysrhythmias can also occur during heart positioning, which may be a result of mechanical irritation or ischemia and can contribute to hemodynamic compromise.

The disturbances typically resolve when the position of the heart is restored. However, if there is ongoing hypotension or low cardiac output despite escalating doses of vasopressors or adequate volume administration, the heart should be returned to its resting position to restore hemodynamics. It should then be determined if there are other options available to support the patient through the grafting process. These may include additional vasopressor support, volume administration, optimization of pH and electrolytes, or administration of amiodarone.

If the patient still does not tolerate positioning, or the hemodynamic disturbances persist despite restoration of heart position, then an alternative surgical strategy may need to be employed. This may include the use of CPB support and/or conversion to a conventional sternotomy.

### 6.4. Postprocedure Intraoperative Management

When grafting is complete, the blood flow through each graft is often measured with transit time flowmetry to ensure adequate flow is present. If there is insufficient flow, the anastomosis can be visualized with the ultrasound probe and revised if necessary. If the flow is satisfactory, protamine is given to reverse the heparin.

If there is no concern for bleeding, one approach is to partially reverse heparin with protamine (e.g., 100 mg) because of concerns about hypercoagulability and graft patency. In our institution we use a full reversal to target the patient’s baseline ACT.

The pericardium is then partially closed. The left lung is reinflated slowly and the LIMA is closely observed to ensure that there is no stretching as the lung expands [58]. Often recruitment maneuvers with sustained positive pressure are required to ensure proper re-expansion, which the surgical team can help guide under direct vision.

Two-lung ventilation is recommenced and bronchoscopy is performed on the left lung. Frequently, mucous and blood can be seen in the left lung, which should be suctioned. If a robot is being used, it is undocked. Chest tubes are placed within the pleura and connected to a drainage system with suction. Typically no chest tube is placed in the pericardium and no epicardial wires are placed. The chest and skin are closed.

### 6.5. Analgesia

Despite relatively small incisions, significant acute pain can still occur after MI-CABG. Thoracotomy for mitral valve repair has been associated with higher chronic postoperative pain than sternotomy [68]. Putative causes include intercostal nerve damage from the thoracotomy, rib and costovertebral joint and muscle injury from the rib spreaders, and pleural irritation from the surgery and chest tube placement [69,70]. Poorly managed acute pain can lead to postoperative pulmonary complications and prolonged ICU and hospital stays [71,72,73]. Additionally, intense postoperative pain is associated with persistent post-surgical pain [71,72]. As such, identifying the most effective approach for postoperative pain management is crucial for patients undergoing MI-CABG procedures.

IV opioids are often considered a primary option for analgesia in cardiac surgery due to their effectiveness in managing acute pain, particularly during and after the procedure. While short-acting IV opioids remain very useful for intraoperative pain management in MI-CABG, providing rapid and controlled analgesia, a multimodal approach to pain management has become favored [74]. This approach combines opioids with non-opioid analgesics and regional anesthesia to minimize side effects and improve patient recovery and is a foundational aspect of enhanced recovery after cardiac surgery [74].

Options for regional techniques include thoracic epidural analgesia (TEA), spinal anesthesia, thoracic paravertebral block (TPVB), erector spinae plane block (ESPB), serratus anterior plane block (SAPB), pectoral nerve (PECS) 2 block, and extrapleural intercostal (ICNB) single-shot or catheter placement and infusion [75].

There is limited evidence available on the use of neuraxial analgesia in MID-CABG procedures. Two small studies demonstrated that patients undergoing MID-CABG had lower pain scores and shorter ICU and hospital stays with continuous TEA (alone or with GA) compared to GA alone, without any reported TEA-related complications [76,77,78].

Studies comparing continuous paravertebral block (PVB) and thoracic epidural analgesia (TEA) with local anesthetic infusion in MID-CABG and TECAB procedures showed similar degrees of postoperative pain control [77,79,80]. No complications were reported with PVB, and only minor issues such as catheter placement failure and transient hypotension were observed with TEA use [77,79,80].

TEA and PVB use in MI-CABG procedures still remains limited given the relative complexity of insertion compared to other techniques and ongoing concern regarding potential side effects including hematoma, hypotension, and respiratory depression [75,77]. However, MI-CABG procedures use lower amounts of heparin compared to those required for CPB which can reduce concerns about bleeding risks relative to traditional CABG.

One study compared the use of a continuous ESPB to conventional multimodal analgesia in MI-CABG patients and found no difference in postoperative opioid consumption [69].

The use of continuous SAPB in MI-CABG has been shown to reduce postoperative pain and opioid use [81,82].

The addition of single-shot SAPB, alone or with PECS-2 blocks, to systemic opioids or multimodal analgesia reduced postoperative pain scores and opioid use compared to systemic therapy alone, although these studies looked predominantly at patients undergoing minimally invasive valve procedures [77].

Compared to single-shot ICNB, continuous extrapleural ICNB catheters provided better post-extubation analgesia in MID-CABG patients [83].

Whilst this is an evolving area of research, regional anesthesia either as the sole approach or in combination with systemic analgesics appears to reduce opioid consumption and provide superior analgesia to IV opioid-based techniques alone [77]. Table 3 summarizes the various regional approaches for MI-CABG procedures.

In a recent practice advisory for the postoperative pain management of cardiac surgical patients, the Society of Cardiovascular Anesthesiologists have recommended consideration of single-shot or continuous SAPB in addition to multimodal analgesics for management of minimally invasive cardiac surgery patients [77].

## 7. Postoperative Management

### 7.1. Extubation

The majority of patients undergoing MI-CABG are suitable for early extubation (<6 h after arrival in the ICU) [84]. If a DLT is being used, it is changed to an SLT in the operating room before transfer. If a bronchial blocker is being used, it is simply removed. The patient is then transferred to the ICU.

Alternatively, it may be suitable to perform on-table extubation (OTE) in the operating room. Although there is still some uncertainty about its benefits in the cardiac surgical population [85], it appears to be a safe and effective strategy in centers where OTE is frequently performed [86,87]. One study identified preoperative characteristics associated with successful on-table extubation in cardiac surgery, including younger age, lower body mass index, absence of chronic lung disease and diabetes, non-full sternotomy, isolated coronary bypass surgery, and elective surgery [88], suggesting OTE is a reasonable approach in MI-CABG patients [86,88,89]. The decision to perform on-table extubation (OTE) in patients undergoing minimally invasive coronary artery bypass grafting (MI-CABG) is made on a case-by-case basis. However, the suggested criteria for safe OTE encompass respiratory, cardiovascular, neurologic, and surgical parameters. The respiratory parameters include adequate oxygenation (PaO_2_ > 75 mmHg with FiO_2_ < 0.4) and effective ventilation (PaCO_2_ < 50 mmHg with spontaneous, unlabored breathing and PEEP < 7.5 cmH_2_O). The neurologic parameters include the absence of residual neuromuscular blockade (train-of-four ratio > 0.9), the patient being awake and responsive to commands without neurological deficits, and adequate analgesia. The cardiovascular parameters include low and non-escalating doses of vasopressors and inotropes and no significant ST segment changes. The metabolic parameters include normothermia (nasopharyngeal or esophageal temperature >36 °C), pH > 7.25, and reasonable urine output. The surgical parameters include minimal chest tube drainage (<100 mL/hr) and no other concerns expressed from the surgical team [90,91,92]. These criteria are summarized in Table 4. Ultimately they should be adapted to individual patients’ underlying physiology, and existing institutional protocols.

### 7.2. ICU Management

All patients should have a chest x-ray on arrival to assess for the presence of a pneumothorax, pleural effusion, lung collapse or consolidation, and proper chest tube, central line, and endotracheal tube placement.

Patients are monitored for signs of ischemia with a 12-lead ECG on arrival, continuous ECG monitoring and serial troponin measurements. ST segment changes and a higher than expected troponin elevation can suggest graft occlusion and may warrant coronary angiography, which if positive would require PCI or return to the operating room for bypass graft revision.

Provided bleeding is minimal, patients are treated with dual antiplatelet therapy in the form of ASA and P2Y12 inhibitors (e.g., clopidogrel) to promote graft patency.

Early mobilization and ambulation are emphasized. If there are no major barriers identified, it is reasonable to encourage patients to sit at the edge of the bed or in a chair on the day of their operation, and to be ambulating on the first postoperative day [93].

Some centers advocate for a “Zero-ICU” protocol after minimally invasive cardiac surgery, where patients are taken to a post-anesthetic care unit (PACU) after surgery and then transferred to a high-dependency unit or ward bed that day [93]. This approach requires careful patient selection, a rigorous perioperative protocol, and a cohesive multidisciplinary team.

## 8. Conclusions

Anesthesia management in MI-CABG is of critical importance in ensuring optimal conditions for surgeons to be able to perform the operation with relative ease, accuracy, and efficiency, thereby achieving excellent patient outcomes, particularly given the unique physiological challenges posed by smaller surgical incisions with limited access and visibility. Integrating these specific surgical conditions with a thorough preoperative assessment, careful intraoperative planning and management, and regimented postoperative care is essential. The anesthesiologist must be familiar with each step of the surgical technique in order to maintain adequate physiology and to expeditiously deal with any problems that arise.

The use of regional anesthesia techniques has become a key element in postoperative pain management, contributing to faster recovery and a reduced incidence of postoperative complications. MI-CABG can allow for early extubation and mobilization, allowing patients to return a functional status swiftly. This depends heavily on the anesthesiologist’s perioperative management. Ultimately, a comprehensive and tailored anesthetic approach allows patients to fully harness the benefits of this surgical approach.

## Figures and Tables

**Table 1:** Surgical approaches to MI-CABG.

Technique	Indications	Surgical Approach	Key Components	Advantages	Limitations
MIDCAB	Isolated proximal LAD disease, hybrid approach for multivessel disease	Small thoracotomy in the 4th or 5th intercostal space	Rib retractor, epicardial stabilizer	Relatively short duration, minimal hemodynamic instability	Limited to proximal LAD disease
MICS-CABG	Multivessel coronary artery disease	Slightly more lateral thoracotomy compared to MIDCAB	Rib retractor, epicardial stabilizer, heart positioner	Useful when multivessel grafting is needed	Longer procedural time, potential for hemodynamic instability
TECAB	Single or multivessel coronary disease	Entirely robotic with multiple endoscopic port sites, no thoracotomy.	Capnothorax, robotic harvest and anastomosis	No thoracotomy incision	Longer procedural time, requires specialized robotic equipment
CPB-assisted MI-CABG	Cases with significant risk of hemodynamic disturbance	Any of the above	Peripheral cannulation, Chitwood clamp or endoballoon if cardioplegia is used	Smooth hemodynamics	Bleeding and coagulopathy

**Table 2:** Contraindications and considerations to MI-CABG by system.

Category	Condition	Contraindication	Considerations
**Cardiac**			
**Coronary Arteries**	Acute LAD occlusion or LAD dissection	Absolute	Unsuitable due to acute nature and complexity.
	ACS with hemodynamic instability	Absolute	High risk due to instability; urgent cases (e.g., unstable angina) may still be candidates.
	Small LIMA (<1.5 mm) or intramyocardial LAD	Relative	Feasibility depends on surgical skill.
	RCA or LCx grafting without suitable targets	Relative	Heart subluxation increases hemodynamic instability risk.
**Echocardiography**	LVEF <20%	Relative	Can be carried out safely with experience but high risk in unstable patients.
	Severe LV dilation (LVEDD >55 mm)	Relative	Same as above.
	Aortic insufficiency	Relative	May preclude CPB-assisted MI-CABG due to cardioplegia delivery issues.
	Significant valvular disease requiring intervention	Relative	May necessitate sternotomy/CPB, though staged TAVI feasible in aortic stenosis.
**Cardiomegaly**	Cardiothoracic ratio >50%	Relative	Complicates surgical access.
**Pericardium**	Inflammatory pericardial disease or adhesions	Relative	Complicates surgical access.
**Respiratory**			
**OLV Suitability**	Severe chronic lung disease	Relative	High risk of hypoxemia on OLV.
**Pleural Disease**	Pleural adhesions	Relative	May increase bleeding and risk of conversion to sternotomy.
**Pulmonary Hypertension**	Severe pulmonary hypertension/RV dysfunction	Relative	OLV and heart manipulation may precipitate RV failure.
**Vascular**			
**Upper Extremity Circulation**	Left subclavian artery stenosis	Absolute	Risk of subclavian steal syndrome; critical due to LIMA-LAD graft reliance.
	Left arm AV fistula	Absolute	Same as above.
**Peripheral Vasculature**	Peripheral vascular disease	Relative	Impacts peripheral CPB feasibility.
**Gastrointestinal**			
**TEE Use**	Esophageal webs, strictures, tumors	Relative	Contraindicates TEE; surgery may proceed without TEE in select cases (e.g., single-vessel MIDCAB).
	Varices, peptic ulcers, hiatal hernia	Relative	TEE risks; discussion if it is safe to proceed without TEE.
**Body Habitus**			
**Musculoskeletal**	Chest wall deformities (e.g., pectus excavatum)	Relative	Complicates access; procedures feasible but challenging.
	Kyphoscoliosis	Relative	Limits positioning and surgical access.
**Obesity**	BMI >30 with surgical access challenges	Relative	Difficult surgical visualization, poor OLV tolerance.

**Table 3:** A summary of regional analgesia approaches in MI-CABG.

Technique	Advantages	Disadvantages	Use During Heparinization
**Deep**			
Thoracic Epidural Analgesia (TEA)	Effective analgesia; potential benefit in recovery	Technical complexity, risk of hypotension, hematoma, respiratory depression, possible catheter placement failure	Caution
Thoracic Paravertebral Block (TPVB)	Fewer complications than TEA; effective analgesia	Same as TEA, but to a lesser degree	Caution
**Superficial**			
Erector Spinae Plane Block (ESPB)	Simpler to perform with lower risk compared to neuraxial techniques	Limited analgesic efficacy compared to multimodal analgesia alone	Safe
Serratus Anterior Plane Block (SAPB)	Simpler to perform with lower risk compared to neuraxial techniques, reduced opioid consumption compared to multimodal analgesia	Catheter placement required, which increases complexity	Safe
PECS II Block	Simpler to perform with lower risk compared to neuraxial techniques, can be performed as a rescue block	Efficacy only demonstrated when combined with other blocks	Safe
Extrapleural Intercostal Nerve Block (ICNB)	Can be performed under direct vision by surgical team after heparin reversal	Single shot less effective than catheter technique	Safe

**Table 4:** Suggested criteria for on-table extubation.

Category	Criterion	Threshold
**Respiratory**	Arterial oxygenation	PaO_2_ > 75 mmHg with FiO_2_ < 0.4
	Ventilation	PaCO_2_ < 50 mmHg with spontaneous, unlabored ventilation; PEEP < 7.5 cmH_2_O
	Breathing pattern	Spontaneous, unlabored ventilation
**Cardiovascular**	Inotropic/vasopressor support	Small and non-escalating doses
	ST segment stability	No ST elevation or significant depression
**Neurological**	Level of consciousness	Awake and able to follow commands
	Neurologic status	No focal neurological deficits
	Residual neuromuscular blockade	Train-of-four ratio > 0.9
	Analgesia	Adequate analgesia
**Metabolic**	Body temperature	>36 °C
	Arterial pH	>7.25
	Urine output	Adequate
**Surgical**	Additional surgical concerns	None
	Chest tube output	<100 mL/h
